# Prophylactic tamsulosin can reduce the risk of urinary retention after surgery in male patients: A systematic review and meta-analysis

**DOI:** 10.3389/fsurg.2022.930707

**Published:** 2022-11-10

**Authors:** Hua Li, Wupeng Zhang, Gaoxiang Xu, Daofeng Wang, Cheng Xu, Hao Zhang, Licheng Zhang, Jiantao Li, Peifu Tang

**Affiliations:** ^1^ Senior Department of Orthopedics, The Fourth Medical Center of Chinese PLA General Hospital, Beijing, China; ^2^ National Clinical Research Center for Orthopedics, Sports Medicine and Rehabilitation, Beijing, China; ^3^ School of Medicine, Nankai University, Tianjin, China

**Keywords:** prophylactic intervention, tamsulosin, postoperative urinary retention, male patients, meta-analysis

## Abstract

**Objective:**

The meta-analysis aimed to estimate the efficacy of prophylactic tamsulosin on postoperative urinary retention (POUR) in male patients.

**Methods:**

Papers were searched in the PubMed, Embase, Web of Science, and Cochrane Library databases with predetermined keywords up to March 1, 2022. The studies reporting the preventive efficacy of prophylactic tamsulosin on POUR among men were identified. Pooled risk ratios (RRs) were calculated based on the random-effects model. Meta-regression was performed to explore potential sources of heterogeneity.

**Results:**

There were 11 studies with 1,046 patients in the tamsulosin group and 1,113 patients in the control group. The risk of POUR was significantly lower in the tamsulosin group (123/1,046 [11.8%] vs. 238/1,119 [19.0%]; RR = 0.61; 95% confidence interval [CI] 0.43 to 0.87; *P* = 0.006; heterogeneity: *I*^2^ = 57%; *P* = 0.009). Administration of tamsulosin was related to higher risk of adverse events (57/688 [8.3%] vs. 33/624 [5.3%]; RR = 1.68; 95% CI: 1.13 to 2.48; *P* = 0.010; heterogeneity: *I*^2^ = 33%; *P* = 0.20). The level of evidence and mean age of the included patients were identified as the potential sources of heterogeneity.

**Conclusion:**

The present meta-analysis indicated that prophylactic tamsulosin helps in preventing POUR and younger patients might benefit more from this preventive regimen. Administrating tamsulosin was also associated with a possibly higher risk of adverse events.

## Introduction

Postoperative urinary retention (POUR) is generally defined as a difficulty to micturate with a full bladder after surgery (1, 2). This condition causes anxiety and is related to poorer patient satisfaction and postoperative outcomes. The published incidence of POUR fluctuates, with a range from 2% to 70% (3–5). Multiple studies have identified the risk factors of POUR (6–8). Male gender is one of the most prominent risk factors of POUR (5, 9–11). The risk of POUR could even be six times higher in men than in women (12). Other risk factors include advanced age (13), nonambulatory surgery (1), and so on. Despite catheterization being the commonly applied practice to treat POUR, such intervention is distressing and can introduce the risk of catheter-related urinary tract infection, urethral trauma, higher hospitalization cost, and delayed discharge (14–16). Therefore, surgeons have been interested in prophylactic interventions, such as pharmacological therapies during the perioperative period, to avoid POUR and the requirements for catheterization.

Alpha adrenergic antagonists such as prazosin, phenoxybenzamine, alfuzosin, and tamsulosin have been permitted in the management of lower urinary tract symptoms secondary to benign prostate hyperplasia (17–19). They can interrupt the pathway at the level of the receptors and then induce relaxation of prostatic smooth muscle to reduce the symptoms. Their off-label uses of POUR are also explored based on the postulation that the postoperatively increased sympathetic activity of the autonomic system plays a major role in the occurrence of POUR (20, 21). In the past several decades, surgeons have studied the prophylactic uses of prazosin, phenoxybenzamine, and alfuzosin. Currently, more studies have focused on super-selective alpha adrenergic antagonists attributing to their less systematic side effects (22).

Tamsulosin is one of the most wildly accepted super-selective alpha adrenergic antagonists. Several studies have estimated the efficacy of tamsulosin on preventing POUR in male patients, but the findings remain controversial. Ghuman et al. conducted a retrospective cohort study in male patients after colorectal surgery and found that prophylactic tamsulosin could reduce the incidence of POUR (23). In the contrast, the randomized control trial (RCT) by Schubert et al. observed no statistical significance in the rate of POUR whether tamsulosin was administrated or not (24). Given that there were also other publications with uncertain results (25, 26), the evaluation of the prophylactic efficacy of tamsulosin against POUR in male patients is still of interest and subject to discussion. The present systematic review and meta-analysis aimed to evaluate the prophylactic efficacy of tamsulosin against POUR in male patients.

## Methods

We conducted this systematic review and meta-analysis following the Preferred Reporting Items for Systematic reviews and Meta-Analyses (PRISMA) Statement protocol (27, 28).

### Search strategy and eligibility

PubMed, Embase, Web of Science, and Cochrane Library databases were searched on 1 March 2022. The search keywords were tamsulosin AND (urinary retention OR voiding difficulty) AND (male OR man). We developed specific search strategies for each database and the references of the identified studies were checked for potential eligibility.

The following inclusion criteria were used:

Publications reporting the preventive efficacy of prophylactic tamsulosin against POUR.
Comparative study design.
Only male patients included in the studies.

We excluded non-English language reports, *in vitro* studies, case reports, brief reports, conference abstracts/posters, or reviews. After removal of duplicates, two authors independently reviewed the titles and abstracts to screen for potentially eligible studies. Full texts were then assessed independently by the same two reviewers to identify the final list of publications suitable for inclusion in the current study. If a disagreement occurred, a third senior doctor was consulted for final assessment and consensus.

### Data extraction

After the final list of included studies was set, data were extracted, including information on the publication, patient attributes, operation, use of the catheter, regimen of tamsulosin, and study design. The primary outcome was the incidence of POUR. Adverse events were also extracted as secondary outcomes. If the necessary information could not be extracted from the original paper, we contacted the corresponding author to request additional information.

### Assessment of quality and bias

The quality of the included studies was assessed independently by two reviewers. For RCTs, the modified Jadad scale was used to assess the methodological quality of randomization, concealment, blinding, and description of withdraws or dropouts (29, 30), and the Cochrane Risk of Bias tool was used to assess the study bias (30, 31). For cohort studies, the Newcastle–Ottawa scale was used to assess the risk of bias (32). The publication bias was estimated by the funnel plot and Peters’ test (33). If a disagreement occurred, a third senior doctor was consulted for final consensus.

### Statistical analysis

Statistical analysis was performed using Review Manager (RevMan, RRID:SCR_003581, version #5.3) and R software (rmeta, RRID:SCR_002270, version #4.1.3), with *P* < 0.05 as the threshold of significance. When comparing the incidence of dichotomous data, such as the rates of POUR or adverse events, risk ratio (RR) was calculated with a confidence interval (CI). Tamsulosin administration was considered a protective intervention if the RR was less than 1. The Mantel–Haenszel (M–H) method was used. We used the *I*^2^ statistic and the *Q* test to measure heterogeneity. The use of a fixed-effects model or random-effects model was based on the assessment of heterogeneity. If *I*^2^ < 50% and the *P*-value for *Q* test > 0.05, the studies were considered minimally heterogeneous and a fixed-effects model was employed for the meta-analysis. A random-effects model was employed when *I*^2^ > 50% or the *P*-value for the *Q* test < 0.05, indicating that there was substantial heterogeneity in the data. Other results suitable for quantitation were presented as a descriptive summary. Sensitivity analyses were conducted using the leave-one-out analyses. Meta-regression was used to identify the potential sources of heterogeneity based on predetermined factors, including the year of publication, mean age of patients recruited, type of operative (urologic surgery or not), type of anesthetic (general anesthesia, neuraxial anesthesia, or mixed) and use of the catheter. As the regimens of tamsulosin differed among studies, we used the total doses of tamsulosin as a potential factor that might influence the heterogeneity. In the first univariate model, each factor was analyzed individually with the effect size and the factors with a *P*-value less than 0.1 were extracted into the next multivariable model. Because studies with different levels of evidence were included in our study, we added the level of evidence (RCT or not) as a potential factor of heterogeneity into the final regression model for adjustment. If a potential factor was confirmed in the final model, a subgroup analysis was performed. If the potential factor was a continuous variable, the bubble plot was used to visualize the relationship between the effect size and the potential factor (34).

## Results

### Overview of search results

**Figure 1:**
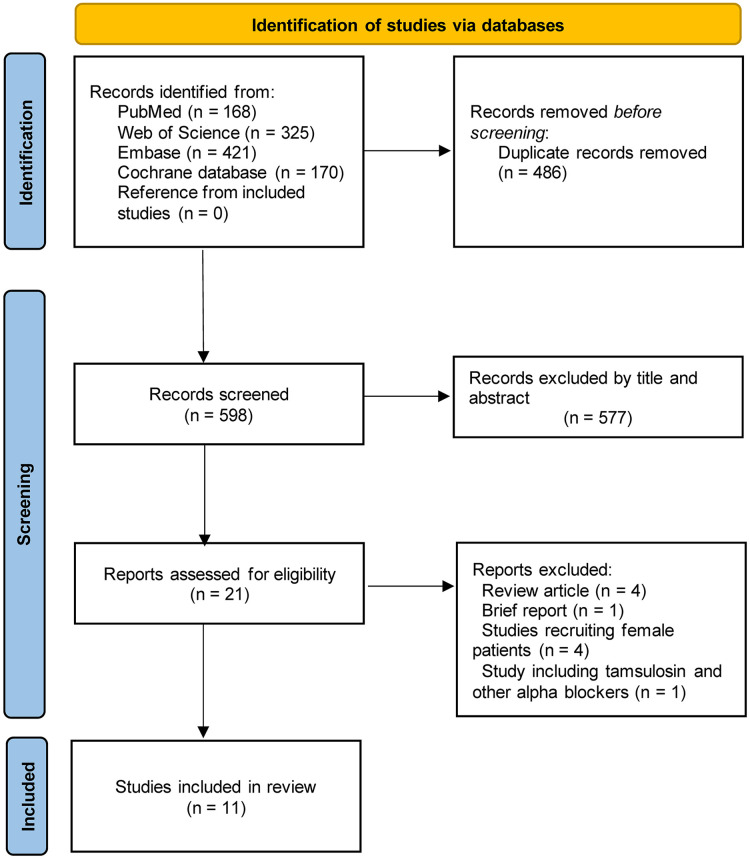
Flowchart of PRISMA.

A total of 1,084 studies were identified in the initial search. After dropping duplicates, we screened the titles and abstracts of 598 papers and then excluded 577 papers that did not meet the criteria. The remaining 21 publications were further assessed by full-text reading and eventually, 9 RCTs and 2 retrospective cohort studies were included (23–26, 35–41) (Figure 1). All of these studies were published in recent 10 years. Nine of the 11 studies reported the data about the use of indwelling catheter after surgery while the other two studies did not provide relevant information. Among the nine studies, five had a relatively high proportion of patients with an indwelling catheter. There were seven types of surgeries, including colorectal surgery, arthroplasty, spinal surgery, herniorrhaphy, varicocelectomy, prostatectomy, and scrotal surgery. We then divided the types of operation into two subtypes based on whether the urologic system was involved. Among the included studies, four indicated that all surgeries were performed under general anesthesia; three indicated that all surgeries were performed under neuraxial anesthesia; three indicated that both neuraxial and general anesthesia were employed; and one did not specify the type of anesthesia. There were 1,046 male patients who received tamsulosin (tamsulosin group) and 1,113 male patients who received placebo or no treatment (control group). In all studies, tamsulosin administration was initiated before surgery. At least two doses of tamsulosin (0.4 mg) were given preoperatively in 818 patients (78.2%) from eight studies. Tamsulosin administration was continued postoperatively in six studies. The details of included studies are summarized in Tables 1, 2.

**Table 1:** Characteristics of included studies.

Authors	Year	Study design	Surgery	Anesthesia	Definition of POUR	Interventions (T/C)
Mohammadi-Fallah et al.	2012	RCT	Herniorrhaphy	Mixed	Inability to void with symptoms	Two doses: tamsulosin 0.4 mg 6 h prior to surgery and 6 h after surgery/placebo.
Madani et al.	2014	RCT	Varicocelectomy Herniorrhaphy Scrotal surgery	Neuraxial	Urine volume retention > 500 ml	Three doses: tamsulosin 0.4 mg; 14 and 2 h prior to surgery and 10 h after surgery/placebo.
Jeong et al.	2014	RCT	Prostatectomy	General	Inability to void postoperatively with symptoms	Daily dose: tamsulosin 0.4 mg; 1 day prior to surgery to 14 days after surgery/no treatment.
Poylin et al.	2015	Retrospective cohort study	Proctectomy	Mixed	PVR > 250 ml or inability to void	Daily dose: tamsulosin 0.4 mg; 3 days prior to surgery/no treatment.
Akkoc et al.	2016	RCT	Urologic surgery	Neuraxial	Urine volume retention > 500 ml	Two doses: tamsulosin 0.4 mg; 14 and 2 h prior to surgery/placebo.
Basheer et al.	2017	RCT	Spinal surgery	General	PVR > 250 ml	Two doses: tamsulosin 0.4 mg; 48 h prior to surgery and the night before surgery/placebo.
Schubert et al.	2019	RCT	Arthroplasty	Mixed	PVR > 200 ml; or urine volume retention > 200 ml with inability to void within 6 h after indwelling urinary catheter removal; or urine volume retention < 200 ml with symptoms and inability to void	Daily dose: tamsulosin 0.4 mg; 5 days prior to surgery, the morning of surgery, and on the first postoperative day/placebo.
Caparelli et al.	2021	RCT	Herniorrhaphy	General	Urine volume retention > 200 ml with inability to void within 6 h postoperatively; or inability to void with symptoms	Single doses: tamsulosin (not giving the dose) 2 h prior to surgery/placebo.
Koukoulis et al.	2021	RCT	Herniorrhaphy	Neuraxial	Inability to void 8 h postoperatively	Two doses: tamsulosin 0.4 mg; 24 and 6 h prior to surgery/placebo.
Rughani et al.	2022	RCT	Spinal surgery	General	Urine volume retention > 300 ml	Daily dose: tamsulosin 0.4 mg; 5 days prior to surgery and 2 days after surgery/placebo.
Ghuman et al.	2022	Retrospective cohort study	Colorectal surgery	NA	According to previous medical records	Daily dose: tamsulosin 0.4 mg; 3 days prior to surgery to the day of discharge/no treatment.

POUR, postoperative urinary retention; T/C, tamsulosin group/control group; RCT, randomized control trial; PVR, post-void residual volume; NA, not applicable.

**Table 2:** Patient attributes of included studies.

Authors	Sample size (T/C) (*n*)	Mean age (y)	Indwelling urinary catheter after surgery (*n*, %)	POUR (T/C) (*n*)	RR (95% CI)
Mohammadi-Fallah et al.	40/40	64.8	0	1/6	0.17 (0.02–1.32)
Madani et al.	118/114	27.6	NA	7/24	0.28 (0.13–0.63)
Jeong et al.	109/109	63.5	218 (100%)	8/19	0.42 (0.19–0.92)
Poylin et al.	30/155	51.2	185 (100%)	2/38	0.27 (0.07–1.07)
Akkoc et al.	60/60	35.6	NA	3/15	0.20 (0.06–0.66)
Basheer et al.	49/46	57.4	81 (85.3%)	16/13	1.16 (0.63–2.13)
Schubert et al.	64/67	61.0	125 (95.4%)	18/24	0.79 (0.47–1.30)
Caparelli et al.	79/90	58.5	0	6/9	1.13 (0.68–1.89)
Koukoulis et al.	51/49	63.5	0	20/17	0.76 (0.28–2.04)
Rughani et al.	245/252	63.6	48 (9.7%)	23/25	0.56 (0.32–1.00)
Ghuman et al.	201/131	65.1	332 (100%)	19/22	0.95 (0.55–1.62)

T/C, Tamsulosin group/Control group; n, number; POUR, postoperative urinary retention; RR, risk ratio; CI, confidence interval; NA, not applicable.

### Assessment of quality and bias

**Figure 2:**
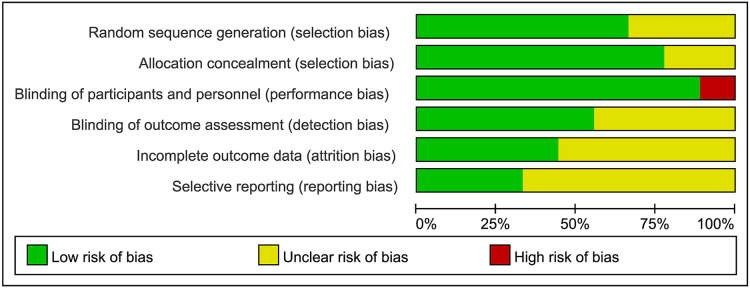
Risk of bias graph.

**Figure 3:**
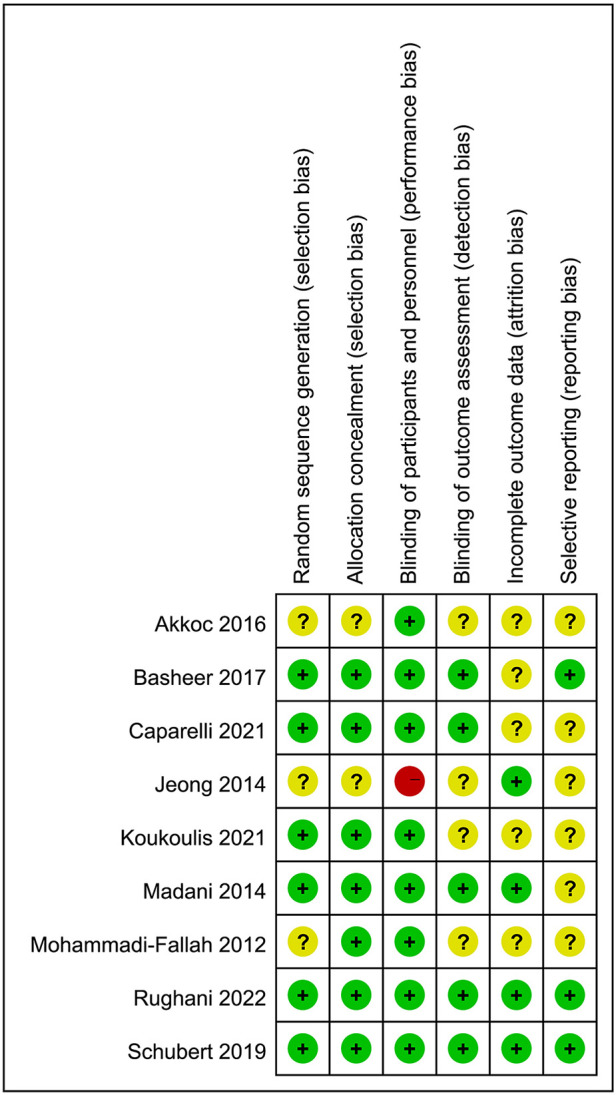
Risk of bias of each research.

**Figure 4:**
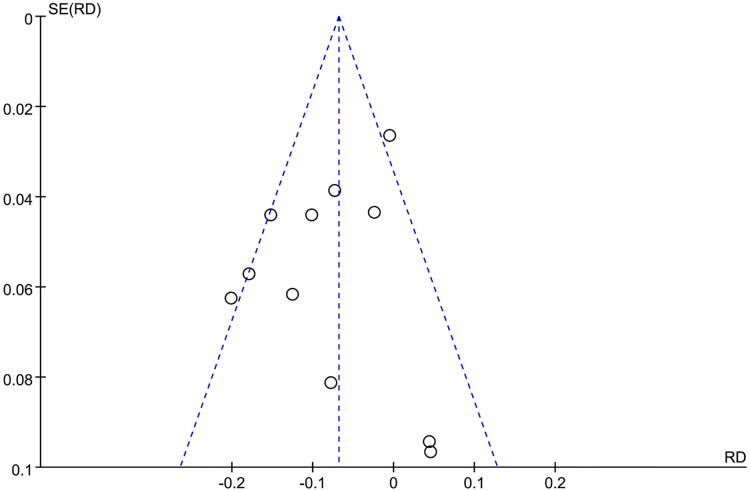
Funnel plot of publication bias.

Most of the RCTs had a Jadad score higher than 4, which indicated that they were of high quality (42). The Jadad score was summarized in Table 3. The risk of bias for RCTs by Cochrane tools was assessed as moderate (Figures 2, 3). The Newcastle–Ottawa rank for two cohort studies represented high quality (Table 4). Overall, the funnel plot did not show the concerns of possible publication bias (Figure 4), which also accorded with the formal test (Peters’ test, *P* = 0.8927).

**Table 3:** Details of Jadad scale.

Authors	Randomization	Concealment	Blinded	Withdraw or drop-out	Total
Schubert et al.	2	2	2	1	7
Basheer et al.	2	1	2	0	5
Caparelli et al.	2	2	2	0	6
Mohammadi-Fallah et al.	1	2	2	0	5
Rughani et al.	2	2	2	1	7
Madani et al.	2	2	2	0	6
Akkoc et al.	1	1	2	0	4
Koukoulis et al.	2	2	2	0	6
Jeong et al.	1	1	0	1	3

**Table 4:** Quality assessment by the Newcastle–Ottawa scale for cohort study.

Study	Selection				Comparability	Outcome		
	1	2	3	4		1	2	3
Ghuman et al.	+	+	+	+	+	+	+	−
Poylin et al.	+	+	+	+	++	+	+	+

Six or more “+” represented a high-quality study.

### Primary outcome

**Figure 5:**
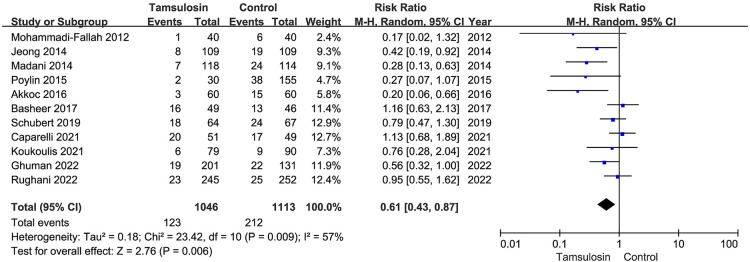
Forest plot for the rate of POUR.

**Figure 6:**
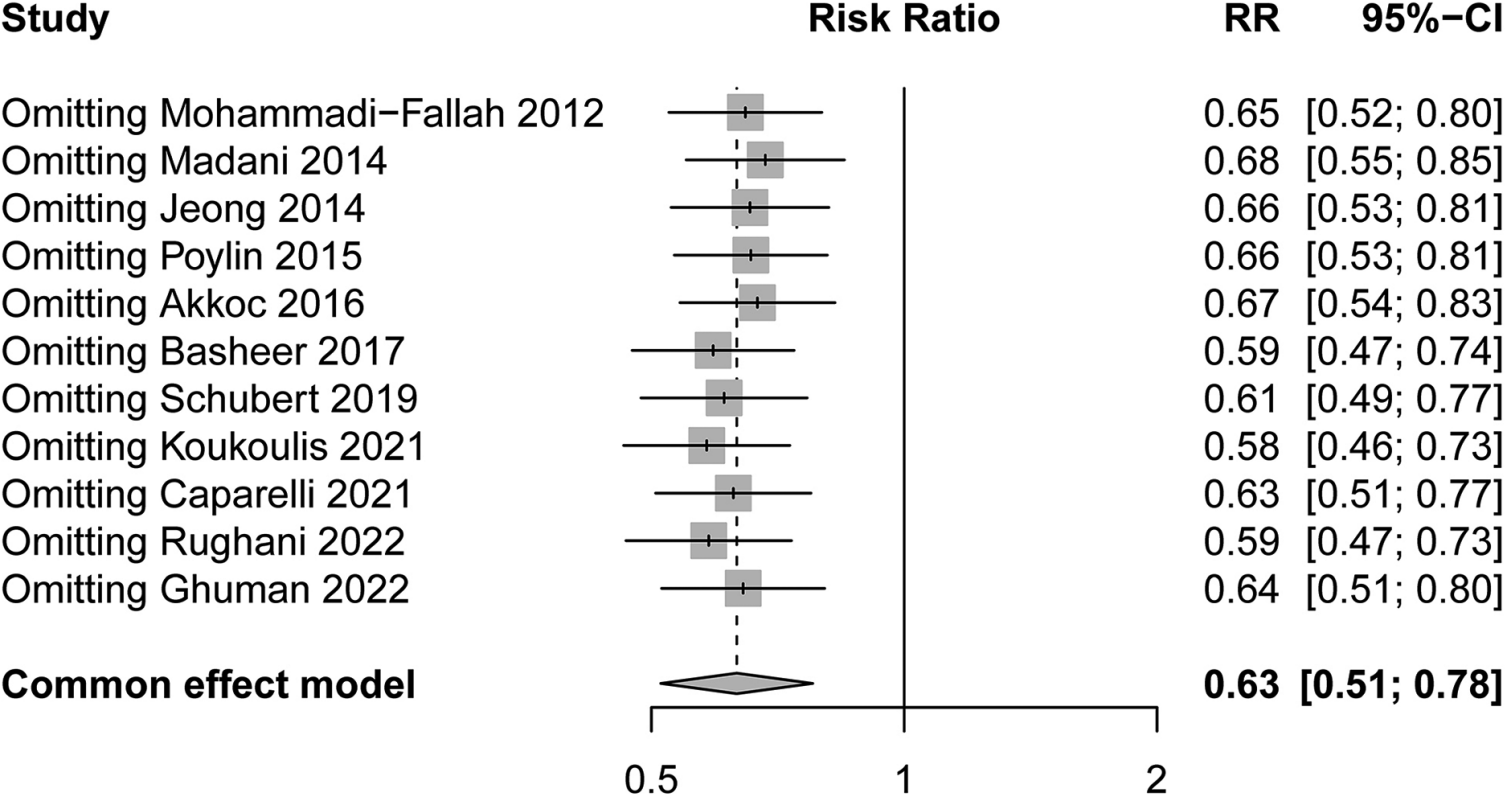
Leave-one-out analysis.

All studies reported the comparison of POUR between the tamsulosin group and the control group. After pooling the data, a total of 335 episodes of POUR were observed. There were 123 patients (11.8%) developing POUR in the tamsulosin group and 238 patients (19.0%) developing POUR in the control group (Table 2). The pooled analysis showed that the administration of tamsulosin was associated with a lower risk of developing POUR (RR = 0.61; 95% CI: 0.43–0.87; *P* = 0.006) with a moderate heterogeneity (*I*^2^ = 57%; *P* = 0.009) (Figure 5). Therefore, the random-effects model was used. The results were robust to the leave-one-out analysis (Figure 6).

### Secondary outcomes

**Figure 7:**
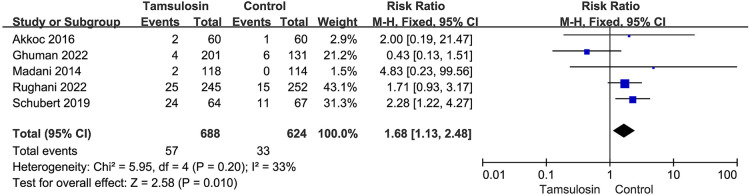
Forest plot for total adverse events.

Five studies reported the data of adverse events, and the pooled analysis showed that tamsulosin administration was associated with a higher risk of adverse events [57/688 (8.3%) vs. 33/624 (5.3%); RR = 1.68; 95% CI: 1.13–2.48; *P* = 0.010; heterogeneity: *I*^2^ = 33%; *P* = 0.20] (23–25, 37, 39) (Figure 7). Dizziness and vomiting were the most frequently described adverse events, with a total of 15 episodes. Schubert et al. reported two cases of floppy iris syndrome in their study, which was considered the most serious adverse event (24).

### Meta-regression

**Figure 8:**
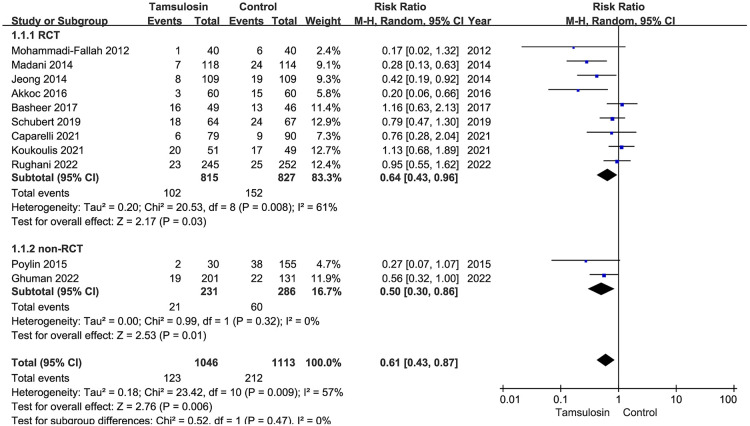
Subgroup analysis of the rate of POUR based on the level of evidence.

**Figure 9:**
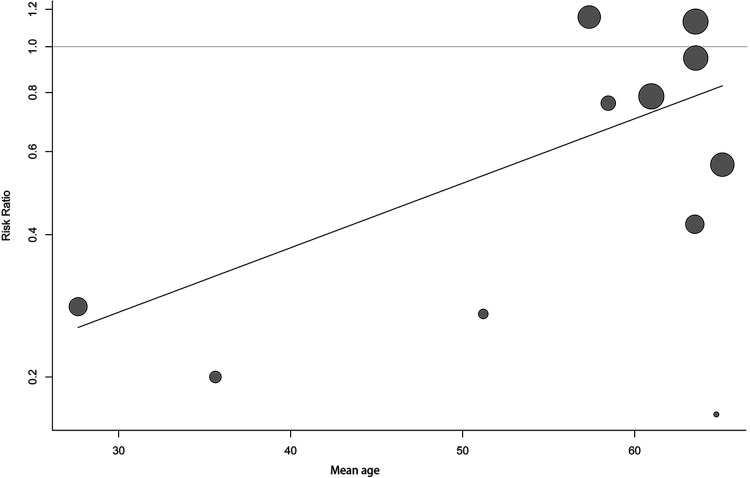
Bubble plot showing the correlation between mean age of patients and risk ratio.

Meta-regression was employed to explore the heterogeneity based on the predetermined factors. At the first step of univariate regression, the year of publication, mean age, and the type of operation were extracted with a crude *P-*value less than 0.1. In the next multiple regression model, the level of evidence and mean age were finally identified as the potential sources of heterogeneity (Table 5). The included studies were then divided into two subgroups based on the level of evidence (RCT or retrospective cohort study). Both of the subgroups supported the preventive efficacy of tamsulosin (RCT group: RR = 0.64, 95% CI: 0.43–0.96; non-RCT group: RR = 0.50, 95% CI: 0.30–0.86). However, the subgroup analysis showed that a large intragroup heterogeneity existed (heterogeneity in the RCT group: *I*^2^ = 61%; *P* = 0.008) and the difference of subgroups were relatively small (heterogeneity between subgroups: *I*^2^ = 0; *P* = 0.47) (Figure 8). The bubble plot showed that a better efficacy of tamsulosin was associated with a lower age (*R*^2^ = 0.652, *P* = 0.0128) (Figure 9).

**Table 5:** Outcomes of the meta-regression.

Factor	Crude coefficient (95% CI)*	*R* ^2^	*I* ^2^	Single *P-*value*	Adjusted coefficient (95% CI)^#^	Meta-regression *P-*value^#^
Publication year	0.1186 (0.0250 to 0.2123)	0.488	41.1%	0.0130	0.0384 (−0.0738 to 0.1506)	0.5022
Mean age	0.0314 (0.0067 to 0.0562)	0.652	32.8%	0.0128	0.0266 (−0.0012 to 0.0545)	0.0610
Operative type (reference: urologic surgery)	−0.7894 (−1.6686 to 0.0899)	0.387	46.3%	0.0785	−0.6459 (−1.4903 to 0.1984)	0.1338
Anesthetic type (reference: general VS mixed anesthesia)	−0.5380 (−1.6843 to 0.6082)	0	66.0%	0.3576	—	—
Anesthetic type (reference: general VS neuraxial anesthesia)	−0.5263 (−1.5719 to 0.5193)	0	66.0%	0.3239	—	—
Total doses of tamsulosin	0.0051 (−0.3109 to 0.3211)	0	73.0%	0.9748	—	—
Use of catheter	−0.4082 (−0.8993 to 0.0828)	0.999	0	0.1032	—	—
Level of evidence (reference: RCT)	—	—	—	—	0.6947 (−1.2944 to −0.0949)	0.0232

CI, confidence interval; RCT, randomized control trial.

^*^ Value calculated in the univariate model.

^#^ Value calculated in the multivariable model.

## Discussion

The present study is the first meta-analysis to evaluate the preventive efficacy of a super-selective alpha adrenergic antagonist, tamsulosin, in male patients at risk of developing POUR. The main administration pattern used in most studies was at least two doses preoperatively. The results showed that prophylactic tamsulosin could reduce the risk of POUR by a statistically significant 39% in a range of surgical procedures. The efficacy might be stronger in younger patients.

POUR is a common issue that occurs in different operation populations with a relatively high prevalence (43, 44). The mechanism of POUR is not yet fully elucidated, while the sympathetic stimulation secondary to pain and surgery is thought to be one of the main contributors (45). Disruption of the sympathetic nervous system at the level of receptors may, therefore, promote micturition and prevent POUR. Based on this theory, alpha adrenergic antagonists have been advocated as a pharmacologic treatment to prevent POUR, which could also reduce the requirements and complications of urinary catheterization. In the past, nonselective alpha blockers such as prazosin and phenoxybenzamine were used to prevent POUR. However, these drugs were limited in clinical use because of concerns about their potential carcinogenicity and cardiovascular effects (19, 46, 47). Tamsulosin is the first super-selective alpha blocker with a preferential selectivity for alpha-1A adrenergic receptors (48), which are located on bladder neck, prostate, and proximal urethra and are likely responsible for contraction (49, 50). The drug has also been used to prevent POUR in several studies, but the results were uncertain. This meta-analysis is of clinical relevance as it integrates the existing evidence to investigate the efficacy of the pharmacological intervention against POUR in male patients.

Our pooled results illustrated that prophylactic tamsulosin could reduce POUR, and these findings were consistent with the physiological and pharmacological mechanisms mentioned above. Two previous meta-analyses evaluated the preventive efficacy of prophylactic alpha blockers on POUR (51, 52). The analysis by Ghuman et al. showed that the risk of POUR could be reduced by almost 50% after the administration of alpha blockers (51). The analysis by Clancy et al. found that the medication could reduce the rate of POUR by 20.6% in patients following inguinal hernia repair (52). Our results revealed a similar trend to their findings. However, their studies included all types of alpha blockers, not only tamsulosin but also those less commonly prescribed nonselective alpha blockers, such as prazosin and phenoxybenzamine. Their pooled data of various pharmacological interventions may introduce bias and heterogeneity in the results. Our review incorporates the consistent studies of tamsulosin, which is one of the most wildly administrated super-selective alpha blockers.

We used the meta-regression to analyze the origins of heterogeneity. The results demonstrated that younger patients might benefit more from prophylactic tamsulosin. Even though the mechanism has not been well clarified, a similar outcome was observed in the study by Roehrborn et al. that tamsulosin might perform better in younger patients (53). They found that additional tamsulosin represented greater improvements in the International Prostate Symptom Score in men with a lower age. In addition, increased age is associated with uncontrolled bladder neck contractions, impaired detrusor contractility, and increased pressure threshold for voiding (1, 7), all of which can jeopardize the efficacy of tamsulosin. Other possible risk factors of POUR have also been explored in our analysis. Previous studies have clarified that the types of anesthesia and operation would influence the development of POUR. The review by Baldini et al. found neuraxial anesthesia at a higher risk of POUR than general anesthesia (6). Postoperative nonambulatory patients may also have a relatively high incidence of POUR at 16%–24%, while the postoperative ambulatory patients have a low rate of 0%–0.8% (1). However, these two factors did not reflect the statistical significance in the meta-regression model. Ghuman et al. identified that preoperative intake of tamsulosin was associated with a strong risk reduction of POUR than postoperative administration (51). In our research, all the included studies launched their initiation of tamsulosin before surgery. Though the protocols of tamsulosin administration were different among studies, the mainstream was two doses of drug between 24 and 48 h before surgery. In order to quantify the regimens of tamsulosin among different studies, we used the value of total doses of tamsulosin as a potential factor in the meta-regression, and the results showed no statistical significance. We also noticed that the timing of drug initiation was highly variable across the included studies, which might also introduce heterogeneity. Because the peak plasma concentration of tamsulosin occurs between 4 and 7 h, it is recommended that administration be initiated at least 24 h before surgery to achieve the maximum clinical effect (54). Another possible source of heterogeneity was the study design. However, the subgroup analysis of RCTs, which represented the integration of the evidence with the highest level, showed that tamsulosin could reduce 36% the risk of POUR, and this result was very close to the pooled calculation of all studies. In the subgroup analysis of RCTs, it should be noted that there still existed moderate heterogeneity. Since the regimens of drug and types of operation differ among studies, we appeal for more studies with RCT study design to better estimate the preventive efficacy of tamsulosin against POUR.

In our research, we summarized the adverse events and found that tamsulosin administration might be associated with a higher risk of adverse events. The most common adverse events were dizziness and vomiting, which might be due to tamsulosin-induced vasodilation (55, 56). We also noticed reports of the serious adverse event, floppy iris syndrome, though it was only reported in one publication with two cases (24). Because alpha-1A adrenergic receptors are also located in the dilator smooth muscle of the iris, tamsulosin may impede mydriasis (57). A cohort study found that patients undergoing cataract surgery might have a 2.3-fold increased risk of floppy iris syndrome with tamsulosin (50). Surgeons should be aware of our assembled evidence, especially when dispensing tamsulosin to patients with cataracts.

Our meta-analysis had several limitations. First, the methodology contained bias due to the possibly unavoidable omission of relevant studies. The sample size was not large enough to avoid potential bias. However, we searched four main databases to identify all comparative studies on the preventive efficacy of tamsulosin against POUR. Based on the available data, we can answer the main questions. Second, the data of POUR were obtained directly from the articles. The definitions of POUR were similar but not identical in these articles, and a few studies did not clarify the definition or evaluation of POUR comprehensively, which could lead to quantitative bias in the data. Third, we did not stratify the analysis by the potential factor, mean age. Because mean age was a continuous variable, we used a bubble plot to reflect the correlation between mean age and risk ratio. However, meta-regression is based on the linear regression theory. We were unable to prove that the model applied was the best fit. We also noted that the subgroup analysis based on the level of evidence might not be rigorous enough attributing to the paucity of publications. Fourth, the regimens of tamsulosin differed among studies. We used the total doses of tamsulosin to estimate their influence on the heterogeneity (Supplementary Figure 1). However, the doses could not represent the regimens of drug thoroughly. In addition, a total of seven types of surgeries were included. In order to explore the influence of operation type on heterogeneity, we used a relatively simple classification in that we divided the studies into two subgroups due to the scarce data in different disciplines. Pooling data based on this classification may introduce bias, though the meta-regression demonstrated that operation type had no significant influence on the heterogeneity. Several steps of the subgroup analysis were simplified to facilitate the feasibility. Thus, the results should be interpreted carefully.

## Conclusion

Our present systematic review and meta-analysis found that prophylactic tamsulosin was related to a 39% reduction in risk of POUR among various operation procedures. Based on available data, at least two doses of tamsulosin before surgery can obtain optimal preventive efficacy. However, a higher risk of adverse events should be aware. This preventive regimen may be more effective in younger patients.

## Data Availability

The original contributions presented in the study are included in the article/**Supplementary Material**, further inquiries can be directed to the corresponding authors.
