# Supplementary figures and images for: Prophylactic tamsulosin can reduce the risk of urinary retention after surgery in male patients: A systematic review and meta-analysis
Source: Front Surg. 2022 Nov 10;9:930707. doi: 10.3389/fsurg.2022.930707 (PMC9684334; doi:10.3389/fsurg.2022.930707)

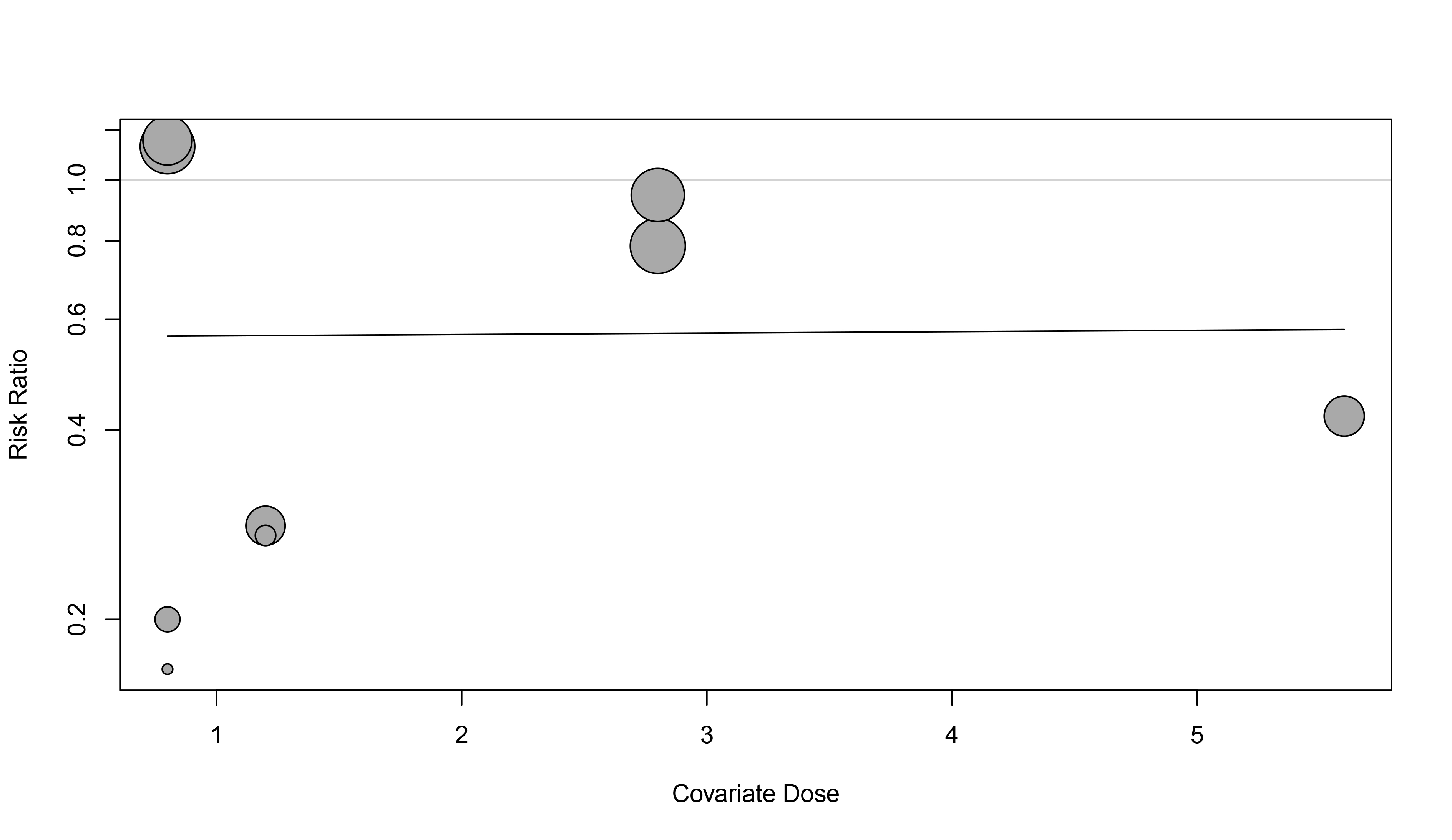

Supplement: Supplementary file 1 [file Image1.jpeg]
